# Characterization of the contributions of Hp-MMP 9 to the serum acute phase protein response of lipopolysaccharide challenged calves

**DOI:** 10.1186/s12917-014-0261-0

**Published:** 2014-10-30

**Authors:** Charles A Hinds, Andrew J Niehaus, Christopher Premanandan, Paivi J Rajala-Schultz, Donald M Rings, Jeffrey Lakritz

**Affiliations:** Department of Veterinary Clinical Sciences, College of Veterinary Medicine, The Ohio State University, 601 Vernon L Tharp Street, Columbus, Ohio 43210 USA; Department of Veterinary Biosciences, College of Veterinary Medicine, The Ohio State University, 1900 Coffey Road, Columbus, Ohio 43210 USA; Department of Veterinary Preventive Medicine, College of Veterinary Medicine, The Ohio State University, 1900 Coffey Road, Columbus, Ohio 43210 USA; Current address: University of Idaho, Caine Veterinary Teaching Center, 1020 East Homedale Road, Caldwell, ID 83607 USA

**Keywords:** Bovine, Neutrophil, Haptoglobin-MMP 9 complexes, Acute phase response, Cortisol

## Abstract

**Background:**

Bovine respiratory disease (BRD) is a costly feature of modern cattle production. Early and accurate detection of BRD may prove useful in the successful management of this disease. The primary objective of the study was to define the time course of covalent complexes of neutrophil, haptoglobin (Hp) and matrix metalloproteinase 9 (Hp-MMP 9) in serum after intravenous lipopolysaccharide (LPS) in comparison to traditional markers. Our hypothesis was that serum concentrations of neutrophil Hp-MMP 9 provides information distinct from traditional acute phase protein markers. To characterize the neutrophil responses to lipopolysaccharide (*E. coli*; O111:B4; 2.5 μg/kg body weight), nine healthy, Jersey calves (65-82 days of age; 74.5 ± 13.1 kg) were challenged and physiologic parameters, peripheral blood cell counts and serum cortisol (C), Hp-MMP 9, Hp, alpha_1_-acid glycoprotein (AGP), serum amyloid A (SAA) were obtained starting 24 hours before to 96 hours post-LPS challenge.

**Results:**

Physiologic parameters (temperature, pulse, respiratory rate) and attitude assessed at each time point indicated that LPS challenge resulted in rapid onset of depression, tachypnea, leukopenia, neutropenia and lymphopenia within 1 hour. Serum C concentrations were significantly increased by 1 hour post-LPS. Serum Hp-MMP 9 complexes were detectable in serum by 0.5 hours and peaked at 16 h, serum total Hp remained <10 μg/mL until 8 hours post LPS infusion and were significantly greater than baseline by 12 hours post-LPS infusion. Serum amyloid A concentrations increased significantly by 8 hours post LPS. Serum concentrations of AGP increased significantly by 16 hours post LPS. Serum concentrations of Hp, SAA and AGP remained significantly greater than baseline out to 96 hours post-LPS. The total systemic exposure to traditional makers is significantly greater than from Hp-MMP 9

**Conclusion:**

Using a well described model for acute phase protein responses, the data demonstrate that serum neutrophil Hp-MMP 9 complexes appear sooner and decline more rapidly than other acute phase proteins (APP). Since Hp-MMP9 is stored pre-formed, it provides information specifically addressing the LPS-induced activation of bovine neutrophils. Contributions of Hp-MMP 9 to the serum acute phase protein response may provide useful information, independent of hepatic responses, in diagnosis of acute inflammation.

## Background

Bovine respiratory disease (BRD) and other acute inflammatory diseases have major impacts on livestock productivity. Recent reports indicate the incidence of BRD morbidity in large feedlots is approximately 5-11% and mortality attributed to BRD is approximately 0.6 – 1.1% [1]. However, 10% morbidity of received animals approximates 1 million head and 1% mortality is >10,000 head [2,3]. While there is some fluctuation in rates of disease, the incidence of BRD remains relatively constant despite application of thoughtful management decisions, development of newer pharmaceutical therapies and biological preventatives. Accurate and early diagnosis of diseases requiring administration of therapeutic agents would be beneficial to cattle industries.

Under experimental and field conditions, the use of APP to detect animals requiring treatment and animals developing lung lesions is useful. For example, haptoglobin (Hp) responses to inflammation in cattle have been evaluated in acute bronchopneumonia [4–7], acute rumen acidosis [8], coliform mastitis [9,10], hepatic lipidosis [11], and transport stress [12,13]. Serum concentrations of Hp in acutely ill cattle increase (>100-fold) reaching maximum concentrations between 48 and 96 h; [14–17] however, serum Hp appears to be a better indicator of clinical responses of calves with BRD to intervention than as a diagnostic for morbidity [5,18]. As the response of a particular APP demonstrate tremendous species and temporal differences, the gradual increase in serum concentrations of Hp over 24-48 hours, while dramatic, appear perhaps less sensitive than other APP in diagnosis of acute disease [1,14]. Other acute phase proteins, such as serum amyloid A (SAA) and alpha 1 acid glycoprotein (AGP) have also been studied in cattle undergoing LPS-challenge and experimental or naturally occurring disease [1,14,19,20]. Several studies indicate that LPS- challenge or experimental bacterial infection elicited earlier increases in serum SAA concentrations, suggesting SAA is more sensitive than Hp due to more rapid production and release [1,14,19,20]. Likewise, studies evaluating AGP after experimental LPS-challenge and *M. hemolytica* A1-challenge demonstrate more marked increases in serum concentrations of Hp and AGP after live bacterial challenge [14]. In contrast, when serum Hp, SAA and AGP were evaluated under field conditions, serum concentrations of Hp were more useful in predicting the presence of respiratory disease and response to therapy, whereas SAA and AGP did not discriminate between animals which became sick and those that did not [21].

In the previous work, we demonstrated that phorbol ester stimulation of isolated peripheral blood neutrophils are is associated with appearance of Hp-MMP 9 complexes in culture medium within 30 minutes [22]. We also identified covalent, heteromeric complexes of Hp in complex with matrix metalloproteinase 9 (Hp-MMP 9), within the serum of cattle with clinically apparent acute onset of septic inflammation of the abdomen or thorax [23]. In these cases, sepsis was associated with the presence of Hp-MMP 9 complexes when serum was analyzed by ELISA. In contrast to free serum Hp, whose main source is the liver during inflammation, serum Hp-MMP 9 complexes are only produced by neutrophils. As such, Hp-MMP 9 complexes, in serum, represent neutrophil degranulation [22].

Intravenous LPS injection has been shown to produce physiologic and biochemical alterations in cattle including increases in APP (Hp [14,24], seromucoid [14], ceruloplasmin [14], α-1 proteinase inhibitor [14], and SAA [24]), decreased feed intake [24–26], increased rectal temperature [24,25,27], dyspnea [24,26,27], increased cytokines (TNFα [24–27], IL-1β [27], IL-6 [27], and IFN-γ [27]), increased cortisol [25,27]. We used this reproducible model to determine the time course of release of Hp-MMP 9 in comparison to other APP (Hp, SAA, AGP) after an acute LPS challenge.

As neutrophils play key roles in the early onset of bovine diseases, we sought to evaluate a biomarker specific to neutrophils for monitoring very early inflammation responses in cattle. The kinetics of most APP involves recognition of pathogens/pathogen products, mediator production and release, gene expression and protein synthesis and release of protein into the circulation [28–32]. Release of neutrophil granule proteins is rapid after *in vitro* stimulation, occurring within 30 minutes of phorbol ester treatment [22]. The objective of the present study was to characterize the time course of serum Hp-MMP 9 complex appearance in relation to changes in the hemogram, serum C concentrations and by comparing with other acute phase proteins in calves (Hp, SAA, AGP).

We propose that Hp-MMP 9 complexes, observed after phorbol ester stimulation of isolated peripheral blood neutrophils *in vitro* and found in acute phase sera, have specific functional significance differing from un-complexed forms of Hp or MMP 9 alone and as a consequence, serum concentrations of Hp-MMP 9 may serve as an independent indicator of clinically important events occurring during acute inflammation.

## Methods

### Experimental design

The following experimental protocol was approved by The Ohio State University, Laboratory Animal Care and Use Committee. The study group consisted of 9 healthy Jersey bull calves between 65-82 days of age (average weight – 75 ± 13 kg; range – 58-100 kg), acclimated to grass hay and 0.7 kg mixed grain diet for 7 days prior to the start of the study. The calves were born at The Ohio State University, Waterman Dairy Farm and were transferred to our facility for use in these studies. Calves were housed in groups of 2 animals, with one group of 3 calves, in climate controlled stalls (average temperature 21.7°C). Physical exams were performed daily throughout the study and body weights were monitored weekly. Twelve hours prior to LPS challenge, calves were fitted with indwelling jugular venous catheters^a^ after clipping the hair and aseptic cleansing the skin using 1% iodine scrub followed by 70% isopropyl alcohol. A 30 cm extension line with infusion port was attached and the catheter was held in place with elastic tape. The catheter was flushed with heparinized saline. Blood was collected from the catheter for CBC and serum biochemical analysis. Serum was collected from clotted whole blood that was centrifuged at 2,000 × g for 20 minutes after clotting at room temperature. The serum was removed and stored at -80°C until analysis.

At time (T) =0 hours, a physical exam was performed and blood was collected for a CBC and for serum collection which was stored for later analysis. All rectal temperature measurements were made using a digital thermometer.^b^ Lipopolysaccharide (LPS),^c^ 2.5 μg/kg body weight, that was diluted in 10 ml of autologous serum and allowed to incubate at 37°C for 30 minutes. After incubation, the LPS solution was administered rapidly via the IV catheter. After administration, each catheter was flushed with 10 mL heparinized saline (10 IU/mL). Physical exams and blood collection were performed at T = -24, 0, 0.5, 1, 1.5, 2, 3, 4, 6, 8, 12, 16, 24, 36, 48, 72, 96 hr post-LPS infusion. After each blood collection, equal volume of normal saline (0.9% NaCl for injection) was administered to maintain blood volume. IV catheters were removed after the last collection time point.

### Serum and blood analyses

All serum analyses were performed using commercially available ELISA kits (Hp, SAA), single radial immunodiffusion (AGP, SRID) according to the manufacturers recommendations, whereas the Hp-MMP 9 ELISA is an in house laboratory assay. Serum C concentrations were determined by use of a solid phase, competitive chemiluminescent enzyme immunoassay and an automated analysis system as described^d^ [33] by an accredited veterinary clinical pathology laboratory. Briefly, aliquots of each calf’s serum were placed into individual test units for analysis. The calibration range of the assay is 28 – 1,380 nmol/L and analytical sensitivity is 5 nmol/L. All samples from each calf were analyzed in a single run.

Serum total Hp was determined using a commercially available bovine Hp ELISA kit.^e^ The analysis was conducted according to the manufacturer’s instructions. Specifically, all serum samples from all calves were diluted 1:2,000 in sample buffer prior to analysis. Serum concentrations were determined from the concentration vs. absorbance relationship of the standard haptoglobin concentrations (7.8-250 ng/mL). All calf serum sample concentrations were corrected for dilution (2,000 fold dilution). Analytical variation between samples on the same day and on multiple days is <8.8 and 12.9% respectively.

Bovine Hp-MMP 9 complexes were determined as described previously [23]. All serum samples were diluted 1:5 with sample diluent (TBS +1% Bovine serum albumin +0.05% Tween 20. After blocking the wells, known concentrations of Hp-MMP 9 (serum, pre-characterized and shown to contain ~912.6 ng/mL Hp-MMP 9) and the LPS challenged calf serum samples were added to wells. If sample absorbance fell outside of the linear portion of the concentration-absorbance line, samples were further diluted to ensure linearity. Between plate variability of calibrators from 5 different plates were less than 3% (median =1.8%; range 0.98-2.7%). The average coefficient of correlation determined from linear regression of the absorbance versus concentration of calibrator was 0.91 (range 0.85 – 0.95). The analytical sensitivity of the assay is 3.5 ng/mL.

Serum concentrations of SAA were determined using a commercial multi-species ELISA (“Phase” Serum Amyloid A assay^f^) used according to the manufacturer’s instructions. All serum samples were diluted 1:500 with sample diluent buffer, and 50 μL of sample or calibrator were added to each well containing the detection antibody and the absorbance determined on a plate reader.^g^ The intra- and inter assay coefficients of variation for the assay were <11% and the analytical sensitivity of the bovine assay is 0.3 μg/mL.

Serum alpha_1_ acid-glycoprotein (AGP) concentrations were determined using a commercial single radial immuno-diffusion assay.^h^ After addition of 5 μL each, of AGP calibrators (1,000 μg/mL, 250 μg/mL, 125 μg/mL) and calf serum samples to individual wells on each plate, the plates were incubated for 48 hours in a humidified container at 37°C. After incubation, the plates were imaged on a light table and the diameter of the rings measured using a 10x scale loupe with metric reticule. The diameter of precipitin rings of calibrators was plotted against the concentration of AGP to obtain an equation of the line. Repeated assay of the calibrators on 27 unique plates, produced coefficient’s of variation between 2.7-3.5% over the range of the calibrators (125-1000 μg/mL), the average coefficient of correlation was 0.997.

### Statistical analysis

Data from the LPS challenge study (temperature, pulse, respiration, WBC count (total WBC, neutrophil counts, band neutrophil counts, lymphocyte counts) differential count, serum concentrations of C, Hp, Hp-MMP 9 complex, SAA, and AGP concentrations) were tabulated by time point and evaluated graphically in a commercial spreadsheet.^i^ After visual comparison of the data, the changes in physiologic variables (temperature, heart rate, respiration), number of peripheral white blood cells, differential counts (total WBC, PMN, band, lymphocyte) and concentrations of the analytes (C, Hp, Hp-MMP 9 complexes, SAA, AGP) over time were examined using PROC MIXED in SAS (v.9.3).^j^ To account for the correlated data structure of the repeated measures from individual calves over time, four covariance structures were tested (compound symmetry, first order autoregressive, heterogeneous first order autoregressive, and unstructured). Time was included in the model as the main variable of interest to evaluate how the different parameters changed in response to the LPS challenge. Baseline (T = 0 hr) was used as the reference level and significance was set at *P* ≤0.05. All measurements at different time-points were compared with the baseline value at T = 0. First order autoregressive covariance structure fitted the data best for SAA, AGP and C as outcomes, compound symmetry covariance structure was used with other outcomes. The areas under the concentration-time (AUC), for each acute phase protein analyte (Hp, Hp-MMP 9, SAA and AGP) were calculated using standard formulae, from time =0 to time =96 hours. No extrapolation of the terminal portion of the curve was conducted. The ratio of AUC_Hp-MMP 9_ (ng*hr/mL), to each of the other analytes (Hp, SAA and AGP) were expressed as a percentage and compared using the Kruskal-Wallis, 1 way ANOVA on ranks with Dunn’s multiple comparison test. Differences in ranks were considered significant when p < 0.05 for each comparison.

## Results

**Figure 1:**
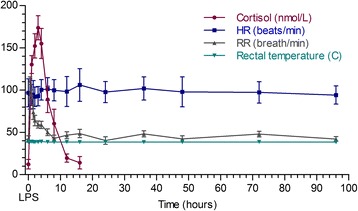
**Mean ± SD heart rate (beats/minute), respiratory rate (breaths/minute), rectal temperarture (°C) and serum C concentrations (nmol/L) observed after intravenous administration of** ***E. coli*** **LPS (O111:B4; 2.5 ug/kg solubilized in autologous serum).** Statistically higher respiratory rates were observed by 0.5 hours post-LPS (p < 0.001) and remained higher than baseline (-24 hour time point) until 6 hours post LPS infusion (p < 0.0034). Serum C values were significantly greater than baseline by 1 hour post LPS (p < 0.0001). There was no statistically significant change in the HR and RT for these calves.

**Figure 2:**
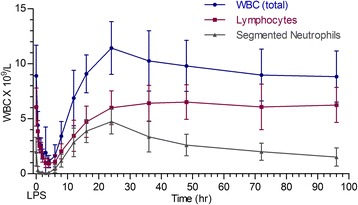
**Mean ± SD total white blood cell counts, lymphocyte count and neutrophil counts in peripheral blood of Jersey calves immediately prior to and up to 96 hours after an intravenous bolus dose of** ***E. coli*** **LPS (O111:B4; 2.5 ug/Kg solubilized in autologous serum).** Plasma WBC were significantly lower than pre-LPS (-24 hr time point) from 0.5 hour – 12 hour time points (p < 00001). Plasma lymphocyte and neutrophil counts showed dramatic drop from the 0.5 hour – 16 hour post LPS challenge time points (P < 0.05).

The most consistent clinical indicators of illness were tachypnea and dyspnea developing within 30 minutes after LPS infusion (p < 0.001 in comparison to T = -24 hours; Figure 1). Respiratory rates remained significantly greater than baseline until 4 h post-LPS and were not significantly greater than baseline by 6 h post-LPS (p = 0.1542; Figure 1). Changes in rectal temperature and heart rate in these calves were minimal and not significantly different from baseline (Figure 1). Significant changes in respiratory rate, occurred with a marked reduction in peripheral leukocytes and rapid increases in serum C (Figure 1). Baseline serum C concentrations were 27.6 ± 7.8 nmol/L (range: 30 – 49.7 nmol/L) at time =0 hours and peaked at 174 ± 40 nmol/L (range: 102-254 nmol/L) by 3 hours post-LPS infusion (p < 0.0001; Figure 1). Total white blood cells (WBC), lymphocytes and neutrophil counts declined from baseline, reaching a nadir at 4 hours (p < 0.0001) post-LPS (Figure 2). Total WBC returned to levels that were not different from baseline by 24-36 hours post-LPS challenge (Figure 2). Peripheral neutropenia was associated with the appearance of “band” PMN at 8 hours (0.2 ± 0.36 × 10 [9]/L; range: 0 – 1.2 × 10 [9]/L; P < 0.0007; data not shown) and remained significantly greater than baseline until 24 hours post LPS (0.66 ± 1.6 × 10 [9]/L; p < 0.0001 compared to baseline; data not shown). Other peripheral leukocyte types (monocytes, eosinophils, basophils) did not change significantly throughout the study period.

**Figure 3:**
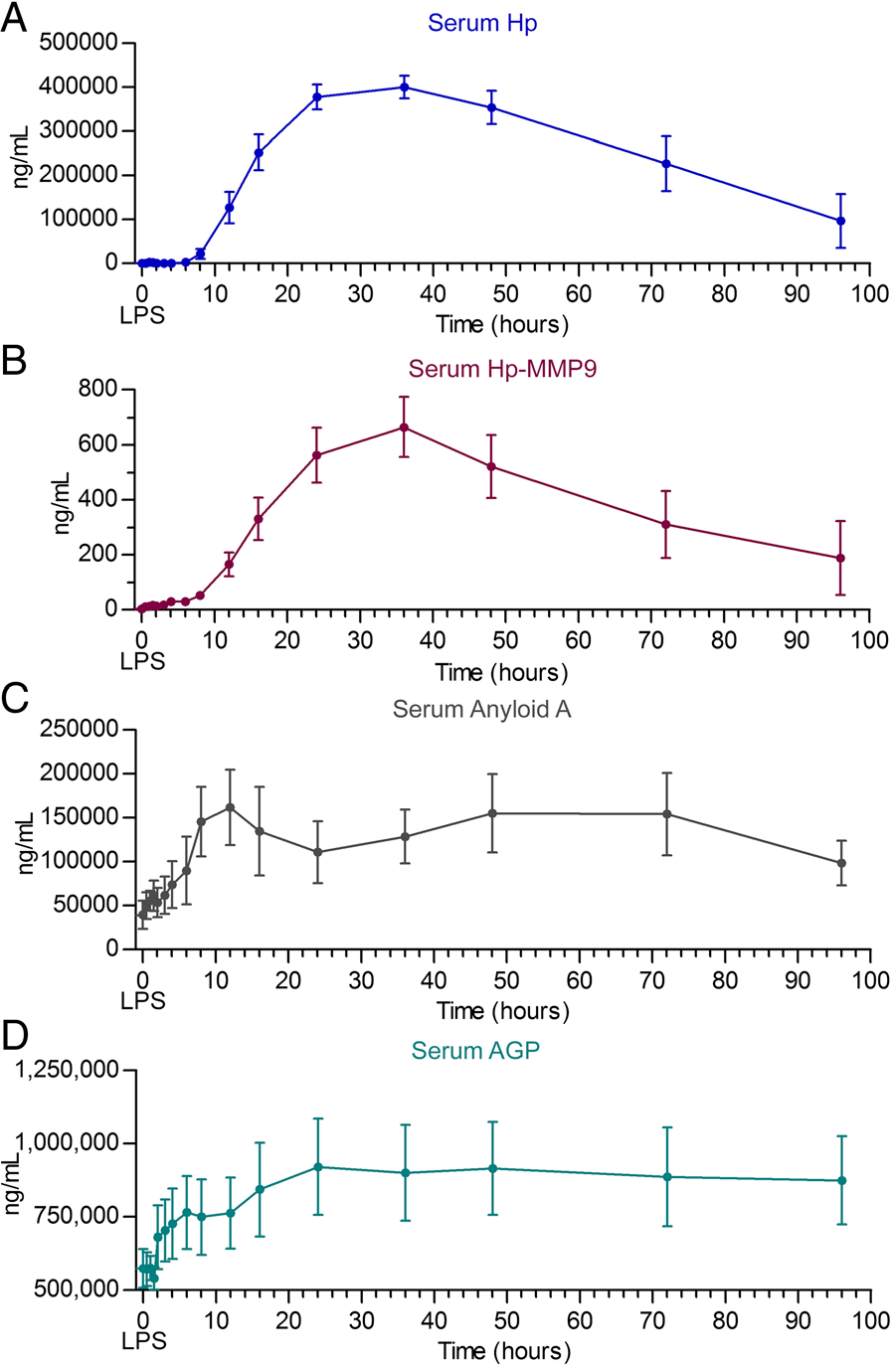
**Mean ± SD of serum concentrations of Hp-MMP 9, total Hp, SA A and serum AGP in peripheral blood of Jersey calves after an intravenous bolus dose of** ***E. coli*** **LPS (O111:B4; 2.5 ug/Kg solubilized in autologous serum).** Concentrations of serum Hp-MMP 9 **(B)** is several orders of magnitude lower than total haptoglobin (Hp) **(A)** and far lower than SAA **(C)** or AGP **(D)**. Serum concentrations of Hp-MMP 9 are in ng/mL. Serum concentrations of Hp, SAA and AGP were converted to ng/mL for comparison to Hp-MMP 9 and as shown, the serum concentrations are reported as logarithm (base 10). Significant increases in serum Hp were detected at 12 hours post-LPS challenge in comparison to pre-LPS time points (-24 hour time points; p < 0.0006) and remained significant through the 96 hour sampling time (p < 0.0087 compared to -24 hour time point). Serum Hp-MMP 9 complex concentrations appeared by 0.5 hrs and were significantly greater than baseline at 16 hour post-LPS challenge (p < 0.008 compared to -24 sample) and returned to baseline values after the 48 hour sampling time (72 hour; p = 0.1254 compared to -24 hour time point). Serum AGP increased to significantly greater than the -24 hour sampling point at 16 hours post-LPS challenge (p < 0.05) and remained significantly greater than baseline through the 96 hour post-LPS time point (p < 0.007). Serum amyloid A concentrations were significantly greater than baseline (-24 hour time point) by 8 hours post-LPS (p < 0.05) and remained greater than baseline through the 96 hour time point (p < 0.05, compared to -24 hour time point).

Administration of a single intravenous dose of LPS resulted in increased serum Hp concentrations. Serum concentrations of total Hp were <10 μg/mL until 8 hours post-LPS infusion (Figure 3A), and increased to significantly greater than baseline by 12 hours post-LPS infusion (127 ± 100 μg/mL; range: 0-368 μg/mL; p = 0.0006) (Figure 3A). Maximum serum concentrations of Hp were 400 ± 73 μg/mL (range: 257-460 μg/mL) 36 hours post-LPS infusion in all 9 experimental animals (Figure 3A). Serum concentrations of Hp remained significantly greater than baseline through the 96 hour time point (p = 0.0087) in 2 of the nine calves; the remaining 7/ 9 calves had serum haptoglobin <30 μg/mL at the 96 hour time point. The increase in serum Hp concentrations occurred after resolution of clinical signs and was associated with return of serum C to baseline concentrations, increasing peripheral leukocyte counts.

Baseline concentrations of AGP were 591 ± 124 μg/mL and did not change significantly until 16 hours post-LPS challenge when serum AGP concentrations were 843 ± 452 ug/mL (p = 0.0434) (Figure 3D). Serum AGP concentrations reached peak levels 24 hours post-LPS (920 ± 466 μg/mL; p = 0.0065). Changes in serum AGP concentration also occurred after clinical signs and serum C concentrations returned to baseline and peripheral blood total WBC and neutrophil counts returned to baseline post LPS (p < 0.0001). Serum concentrations of AGP remained >800 μg/mL throughout the remainder of the study period.

Serum concentrations of SAA responded to LPS challenge increasing from 29 ± 35 μg/mL at baseline to 162 ± 121 μg/mL; 12 h post-LPS challenge; p = 0.0049), and remained significantly greater than baseline at 96 hours post-LPS infusion (98.4 ± 72 μg/mL; p = 0.03) (Figure 3C). Like serum concentrations of Hp and AGP, changes in SAA were significantly greater than baseline by 8 hours and did not correspond to changes in clinical signs, serum C and peripheral WBC counts. Serum amyloid A concentrations peaked 12 hours post LPS, and remained above baseline concentrations through the end of the study.

Serum concentrations of Hp-MMP 9 complexes were detectable (>3.5 ng/mL) from 0.5 – 12 hours post-LPS, although these values were not significantly different from baseline (p = 0.82) (Figure 3B). Serum concentrations of Hp-MMP 9 continued to increase until 36 hrs post-challenge, reaching concentrations of 665 ± 308 ng/mL (range 298-1311 ng/mL; p < 0.0001). Unlike the other APP markers (Hp, SAA, AGP), serum concentrations of Hp-MMP 9 decreased to concentrations that were not significantly different from baseline by 72 hours post-LPS infusion (p = 0.1254). Serum concentrations of Hp-MMP 9 were <55 ng/mL in 7/9 calves at 96 hours.

**Figure 4:**
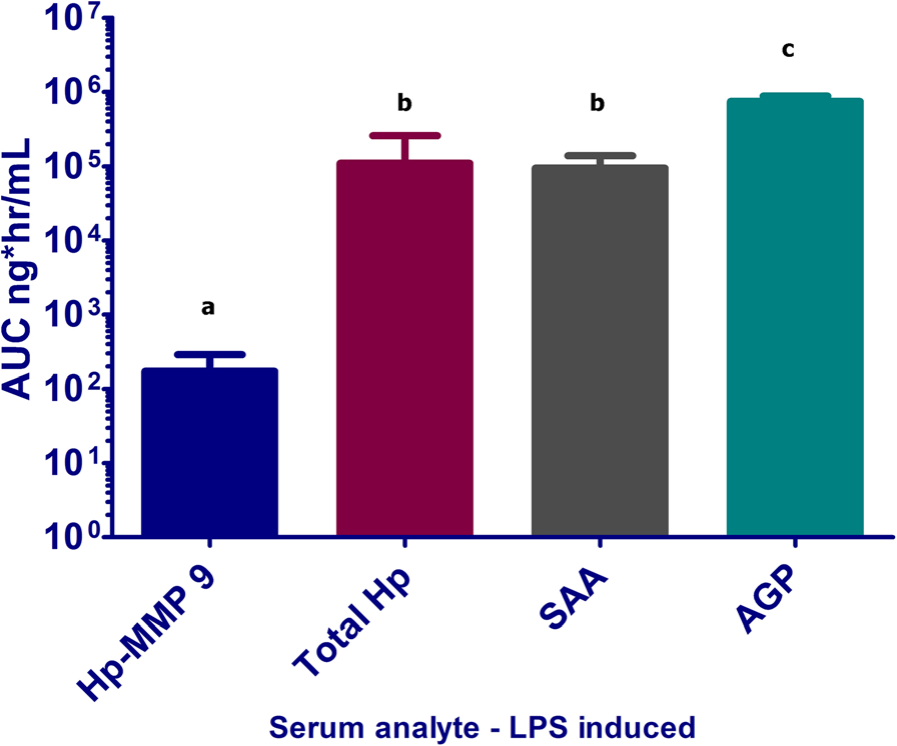
**Total systemic exposure of animal to serum acute phase proteins. Serum concentrations of serum acute phase proteins over time were converted to ng/mL and the area under the curve was calculated using the trapezoidal rule.** Data for each analyte are reported as AUC_0-96 hr_ and plotted on log-linear graph. Area under the curve of serum Hp-MMP 9 was significantly lower than all 3 other serum acute phase proteins (p < 0.01) when evaluated using Kruskal-Wallis, 1 way ANOVA on ranks with Dunn’s multiple comparison test. There was no significant difference between total serum Hp and SAA over the time course of analysis and both SAA and Hp had significantly lower exposure in comparison to the areas observed for AGP. Differences in the letters over bars indicate significant differences between AUC for each analyte.

The average exposure (AUC), to LPS induced serum acute phase proteins demonstrated that exposure (concentration × time) to Hp-MMP 9 differed significantly from that of Hp (p < 0.05) and AGP (p < 0.001), but not SAA Figure 4). Area under the curve for Hp-MMP 9 (AUC_Hp-MMP 9_) was 0.14 ± 0.05% of that for Hp, 0.13 ± 0.06% of that for SAA and 0.05 ± 0.03% of that for AGP (P < 0.01 for Hp-MMP 9 compared to Hp and AGP responses; Figure 4). The AUC_AGP_ was significantly greater than AUC_SAA_ (p < 0.01).

## Discussion

The purpose of this study was to evaluate the time course of a neutrophil biomarker (Hp-MMP 9 complexes) after LPS challenge in calves in comparison to responses of established serum APP markers in cattle. Using a single challenge stimulus producing consistent clinical, hematologic, physiologic and APP responses, the concentration versus time profile of this neutrophil biomarker may prove useful to define the activation of neutrophils early in the course of inflammation. While not fully replicating the major clinical and pathologic findings associated with natural BRD, the characteristics of the LPS stimulus should allow dissection of neutrophil from other APP responses occurring in inflammation [27,34,35]. In clinical terms, we believe early and specific detection of neutrophil activation should provide an indication of early responses to infection. Further, this response is distinct from that of the liver [36].

Bovine neutrophils have been shown to play key roles in host responses to infection [34]. Experimental bacterial pneumonia models have demonstrated rapid recruitment of neutrophils to the lung is associated with tachypnea, dyspnea, hypercortisolemia, peripheral leukopenia and influx of neutrophils into the lungs [27,34,35,37–40]. Our previous studies demonstrated a unique form of neutrophil matrix-metalloproteinase 9, covalently linked to haptoglobin (Hp-MMP 9) are stored in and released by neutrophils; and these complexes are present within the serum of cattle with acute, polymicrobial sepsis [22,23]. Therefore, as a unique marker produced by the neutrophil, serum Hp-MMP 9 complexes should herald early changes in neutrophils associated with the host response to inflammation.

Both natural and experimental infection studies demonstrate the appearance of APP supporting their use in detection of inflammation and response to therapy [1,14,18,19,21,24,41–47]. Although some APP have proven useful in the evaluation of illness in cattle, their contributions to the bovine serum acute phase response, represent contributions of hepatocytes or other tissues in response to proximate mediators produced by other cells [1,14,44,45]. Timing of APP responses vary by APP marker and do not necessarily represent events occurring in the sub-clinical phase of disease such as when changes in clinical signs, serum C and peripheral leukopenia occur. Upon activation, peripheral blood neutrophils are a source of many proteins, including matrix metalloproteinase 9, Hp and AGP. These neutrophil APP in serum are a part of the acute phase proteome; however, current assays do not identify sources of these proteins [48–50].

As a component of neutrophil granules, Hp-MMP 9 should not be present within the circulation of healthy animals and prior studies demonstrated Hp-MMP 9 was not present in the serum of healthy cows [23]. After an intravenous dose of LPS, serum concentrations of Hp-MMP 9 were above the limit of detection of our ELISA (>3.5 ng/mL) by 1 hour. Serum Hp-MMP 9 complexes are detected when changes in respiratory rate, serum C and WBC numbers occur. We have also demonstrated cattle undergoing experimental bacterial pneumonia and naturally occurring cases of acute poly-microbial sepsis were associated with increased serum concentrations of Hp-MMP 9 complexes [23,24]. Experimental bacterial infection is associated with lung lesions consistent with *Mannheimia hemolytica* infection in these calves and with rapid increases in serum concentrations of Hp and Hp-MMP 9 complexes [51]. Similarly, transportation is associated with increased incidence of respiratory disease and Hp is proposed to be a marker of non-inflammatory stress [52,53]. Stress induced induction of serum Hp still involves release of production of cytokines and hepatic expression of Hp and other APP. [53] A recent study demonstrated the appearance of Hp-MMP 9 in serum after transportation suggesting transportation stress is also associated with measurable responses of neutrophils [52,54]. These studies support the further evaluation of Hp-MMP 9 complex as a marker of early inflammation.

Both neutrophil Hp-MMP 9 and SAA showed rapid increases in serum concentrations in LPS challenged calves, consistent with earlier studies [1,19,45,55]. However, individual calf data for SAA varied somewhat, making them significantly different from baseline only by 8 hours post-LPS. However, serum Hp-MMP 9, and SAA concentrations observed after LPS challenge increased concurrently with onset of tachypnea, peak serum C concentrations and reduction of circulating leukocytes. Our results are consistent with these findings; however, we observed sustained SAA concentrations to at least 96 hours post-LPS challenge [1,19,27,43,45,55,56]. Other studies conducting i.v. LPS challenge, characterized the induction of SAA out to 10 hours post-challenge [1,27,45]. Prolonged elevations of SAA observed in our study suggest continued production after LPS stimulation. It is plausible then, that use of SAA in animals may be useful in diagnosis of acute inflammation. However, most of the SAA produced in association with LPS-induced inflammation also represents hepatic responses [56].

In contrast, the clinical, hematologic and C responses occurring in our calves after intravenous LPS challenge preceded increased serum Hp and AGP concentrations by several hours. Serum Hp concentrations remained normal until 8 hours; however, 5/9 had no detectable Hp until 12 hours post LPS challenge. Similarly, serum AGP concentrations were observed to increase significantly at 16 hours post LPS. As previous studies demonstrate, serum Hp and AGP concentrations remain significantly greater than baseline until the end of the study period, well after the LPS induced changes in respiratory function, serum C and leukopenia had resolved [18–20]. Like Hp, Hp-MMP 9, AGP is also produced by bovine neutrophils [49]. It is plausible that serum AGP concentrations as detected with currently available methods may reflect contributions from the neutrophil. However, these methods do not specifically identify this form autonomously of others present.

Serum APP terminal half-life is been proposed to be useful in terms of effectiveness of empirical antimicrobial therapy for pneumonia in humans [57]. The serum concentration versus time profile of Hp-MMP 9 (AUC) was smaller than that of other APP measured. Area’s under the Hp-MMP 9, concentration-time curve (AUC_HP-MMP 9_) in our calves were 0.14% of that measured for serum total Hp (AUC_HP_), 0.13% of AUC_SAA_ and 0.05% of AUC_AGP_. This suggests that the animal’s exposure to Hp-MMP 9 is much lower than that for Hp, SAA and AGP. This seems plausible since neutrophils and their granule proteins are limited in comparison to hepatic APP induced by inflammatory mediators. Characterization of the elimination processes of each APP may help to define the half-life of these proteins after induction. This information may provide a means for evaluation of the rate of normalization associated with therapy.

## Conclusions

As Hp-MMP 9 is detectable early after a consistent inflammatory stimulus when animals develop clinical signs, we believe that like SAA, it may serve as a useful marker of early inflammation. The serum content of this neutrophil protein complex induced by inflammation is limited in extent and is masked by APP produced by the liver. Dissecting the contribution of the neutrophil to the APP responses produced by other sources may prove useful as an adjunct to the clinical examination after arrival at the feedlot, for pre-slaughter exams and other situations. The availability of a test providing objective data regarding the status of the neutrophil, so intimately involved in acute inflammation, may have potential in clinical algorithms and decision making used in diseased cattle.

## Endnotes

^a^Angiocath, 16GA, 3.5 inch; Becton Dickinson, Sandy, Utah

^b^GLA M700 Digital Thermometer; GLA Agricultural electronics, San Luis Obispo, CA 93401

^c^*Escherichia coli* O111:B4; L2630 Sigma-Aldrich, St. Louis, MO.

^d^IMMULITE 1000 Cortisol, Immunoassay system. DPS, Los Angeles, CA

^e^Life diagnostics, Haptoglobin ELISA test kit; catalogue #2410-7. West Chester, PA 19380; www.lifediagnostics.com

^f^Multispecies Cat no. TP-802; Tri-Delta Development LTD, Kildare, Ireland

^g^Labsystems, Multiskan MS; P97180; Vienna, VA, 22182

^h^Cardiotech services, P0101-1; Louisville KY 40205

^i^Microsoft Excel, Microsoft, Corp. Redmond, WA 98052-6399

^j^SAS, Version 9.2, SAS Institute, Cary NC 27513-2414; www.sas.com

## Abbreviations

AGP: Alpha 1 acid-glycoprotein
ANOVA: Analysis of variance
APP: Acute phase protein
AUC: Area under the curve
BRD: Bovine respiratory disease
C: Cortisol
ELISA: Enzyme linked immunosorbent assay
Hp: Serum total haptoglobin concentration
Hp-MMP 9: Hp in complex with matrix metalloproteinase 9
LPS: Lipopolysaccharide
NaCl: 0.9% sodium chloride for injection
PMN: Neutrophil
SAA: Serum amyloid A
TBS: Tris buffered saline
WBC: White blood cell
